# Machine Learning for Opportunistic Screening for Osteoporosis from CT Scans of the Wrist and Forearm

**DOI:** 10.3390/diagnostics12030691

**Published:** 2022-03-11

**Authors:** Ronnie Sebro, Cynthia De la Garza-Ramos

**Affiliations:** 1 Mayo Clinic Florida, Department of Radiology, Jacksonville, FL 32224, USA; delagarza-ramos.cynthia@mayo.edu; 2 Center for Augmented Intelligence, Mayo Clinic Florida, Department of Radiology, Jacksonville, FL 32224, USA

**Keywords:** computed tomography, CT attenuation, metacarpal, radius, ulna, scaphoid, lunate, triquetrum, pisiform, trapezium, trapezoid, capitate, hamate, DEXA, bone mineral density

## Abstract

**Background**: We investigated whether opportunistic screening for osteoporosis can be done from computed tomography (CT) scans of the wrist/forearm using machine learning. **Methods:** A retrospective study of 196 patients aged 50 years or greater who underwent CT scans of the wrist/forearm and dual-energy X-ray absorptiometry (DEXA) scans within 12 months of each other was performed. Volumetric segmentation of the forearm, carpal, and metacarpal bones was performed to obtain the mean CT attenuation of each bone. The correlations of the CT attenuations of each of the wrist/forearm bones and their correlations to the DEXA measurements were calculated. The study was divided into training/validation (n = 96) and test (n = 100) datasets. The performance of multivariable support vector machines (SVMs) was evaluated in the test dataset and compared to the CT attenuation of the distal third of the radial shaft (radius 33%). **Results:** There were positive correlations between each of the CT attenuations of the wrist/forearm bones, and with DEXA measurements. A threshold hamate CT attenuation of 170.2 Hounsfield units had a sensitivity of 69.2% and a specificity of 77.1% for identifying patients with osteoporosis. The radial-basis-function (RBF) kernel SVM (AUC = 0.818) was the best for predicting osteoporosis with a higher AUC than other models and better than the radius 33% (AUC = 0.576) (*p* = 0.020). **Conclusions:** Opportunistic screening for osteoporosis could be performed using CT scans of the wrist/forearm. Multivariable machine learning techniques, such as SVM with RBF kernels, that use data from multiple bones were more accurate than using the CT attenuation of a single bone.

## 1. Introduction

Bone mineral density (BMD) decreases with age, with the decrease being more evident and rapid in post-menopausal females [1,2,3]. Decreased BMD increases the risk of frailty fractures including fractures of the spine, forearm, and hips [4,5,6,7,8]. Fractures of the hips are associated with increased 1-year mortality following the fracture of approximately 15–36%, therefore identifying risk factors for frailty fractures and hip fractures is of increased clinical importance [9,10]. Dual-energy X-ray absorptiometry (DEXA) is the gold standard screening test for the evaluation of BMD [11]. DEXA evaluates BMD in the L1–L4 lumbar spine, total hip, and femoral neck and compares these values to those of normal young adults (20–29 years of age) from the National Health and Nutrition Examination Survey (NHANES) III cohort to create BMD T-scores [12,13]. The World Health Organization (WHO) and the International Society for Clinical Densitometry (ISCD) have guidelines for the classification of patients aged 50 years or greater with low BMD as osteoporotic or osteopenic based on DEXA BMD T-scores. Patients aged 50 years or greater with lowest BMD T-scores ≥ −1 have normal BMD, patients aged 50 years or greater with −2.5 ≤ lowest BMD T-scores < −1 have osteopenia, and patients aged 50 years or greater with lowest BMD T-scores ≤ −2.5 have osteoporosis [14]. The University of Sheffield Fracture Risk Assessment Tool (FRAX) utilizes patient age, sex, femoral neck BMD, and other variables to predict the risk of future femoral fracture [15]. Trabecular bone scores (TBS) are based on textural analysis of bone microarchitecture, and TBS in conjunction with BMD scores were shown to improve the overall predictive properties of DEXA measurements for future fractures [16,17].

Opportunistic screening for osteoporosis was performed using data from computed tomography (CT) scans of the chest/thorax [18], abdomen and pelvis [19,20], and cardiac CT [21]. A strong correlation was noted between the CT attenuation of trabecular bone in the thoracolumbar spine and BMD measurements [18,19,20,21]. Because forearm frailty fractures are associated with low BMD, we hypothesized that the CT attenuation of the wrist and forearm bones would correlate with BMD measurements. A recent study showed that the cortical thickness of the second metacarpal is also correlated with DEXA BMD measurements [22]. Therefore, we hypothesized that the CT attenuation in the metacarpals may also be correlated with BMD DEXA measurements. We hypothesized that routinely obtained CT scans of the wrist/forearm bones in patients aged 50 years or greater could be used to provide insight into whether a patient has osteoporosis or osteopenia and whether the patient needs to be subsequently screened using DEXA.

The aims of this study were (1) to evaluate the correlations between the CT attenuation of the wrist/forearm bones (distal third of the radius; ultradistal radius (radius UD) (distal radial metaphysis and epiphysis), distal third of the radius shaft (radius 33%), distal third of the ulna, ultradistal ulna (ulna UD) (distal ulnar metaphysis and diaphysis), distal third of the ulnar shaft (ulna 33%), scaphoid, lunate, triquetrum, pisiform, trapezium, trapezoid, capitate, hamate, and proximal thirds of the first through fifth metacarpals) in men and women; (2) to evaluate the correlations between CT attenuation of the wrist/forearm bones and L1–L4 BMD, L1–L4 BMD T-score, L1–L4 TBS, total hip BMD, total hip BMD T-score, femoral neck BMD, and femoral neck BMD T-score; (3) to evaluate the predictive performance of each wrist/forearm bone regarding predicting (i) osteoporosis, (ii) osteopenia/osteoporosis, (iii) femoral neck BMD ≤ −2.5, and (iv) femoral neck BMD < −1; and (4) to use machine learning to identify the best combination of clinical/demographic variables and the CT attenuation of wrist/forearm bones to predict (i) osteoporosis, (ii) osteopenia/osteoporosis, (iii) femoral neck BMD ≤ −2.5, and (iv) femoral neck BMD < −1.

## 2. Materials and Methods

The study was compliant with the Health Insurance Portability and Accountability Act of 1996 (HIPAA), and the study protocol was reviewed and approved by the local Institutional Review Board (IRB) at a tertiary care academic medical center. The need for signed informed consent from each patient was waived by the IRB. The work described was carried out in accordance with The Code of Ethics of the World Medical Association (Declaration of Helsinki) for experiments involving humans.

### 2.1. CT Scanner Protocol and CT Attenuation Measurements

CT scans were performed on Siemens Somatom Definition Edge and Siemens Flash (Siemens Healthineers, Erlangen, Germany) scanners. CT scans were performed without intravenous contrast at 120 kVp, 200–250 mA, field of view (FOV) 118–120 mm, tilt of 0° in the axial plane at 0.4–0.5 mm, and reconstructed in the coronal and sagittal planes at 1 mm.

### 2.2. DEXA Scanners

DEXA scans were performed using General Electric (GE) (Waukesha, WI, USA) Luna iDXA Scanners.

The study cohort comprised patients aged 50 years or older, who were evaluated and/or treated between 1 January 2015 and 30 September 2021. Patients were included if they had CT scans of an upper extremity, including the wrist and forearm, and DEXA scans (of the lumbar spine and hips) within 12 months of each other. Patients were excluded if they had prior surgery of the wrist/forearm, hips, or spine with implantation of hardware that would affect CT attenuation measurements.

### 2.3. Segmentations

**Figure 1:**
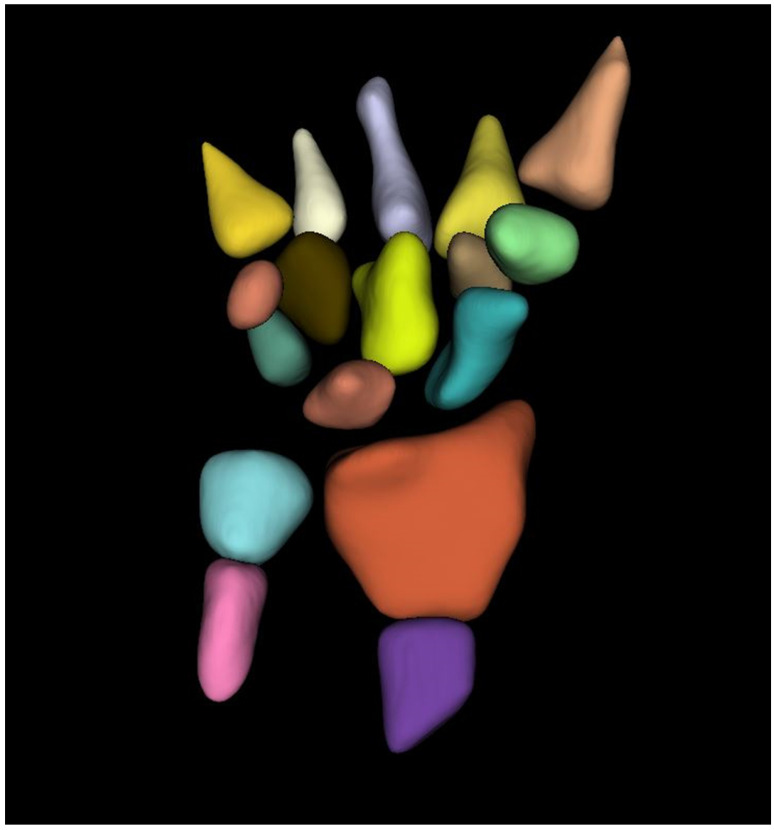
Semi-automated volumetric segmentation mask of the bones of the wrist and forearm. Coronal mask of the wrist/forearm demonstrating segmentation of the distal radius, ulna, carpal bones, and bases of the metacarpals.

The trabecular component of each bone was carefully, manually segmented using 3D-Slicer [23]. Care was taken to avoid bone lesions (hemangiomas and bone islands), osteophytes, and the bony cortex. No specific attenuation threshold was used to segment the trabecular bone. The subcortical regions were segmented manually and then automatically interpolated between CT slices. The entire volume of each bone was segmented, and the mean CT attenuation of each bone was recorded (Figure 1).

We calculated the CT attenuation of the distal third of the radius, radius UD, radius 33%, the distal third of the ulna, ulna UD, ulna 33%, scaphoid, lunate, triquetrum, pisiform, trapezium, trapezoid, capitate, hamate, and proximal thirds of the first through fifth metacarpals. Segmentations were performed by a trained research assistant (3 weeks of training doing all 3D Slicer tutorials; 4 months of experience with 3D Slicer doing similar projects before doing the current project) and were reviewed by a fellowship-trained musculoskeletal radiologist with 10 years of experience.

### 2.4. Statistical Analysis

A sample size of 100 patients was determined to have 80% power with a type I error rate of 5% to detect a difference between two receiver operator characteristic (ROC) curves if the first ROC curve has an area under the receiver operator characteristic curve (AUC) of 0.80 and the second ROC curve has an AUC of 0.60, and there are 74% controls and 26% cases. Therefore, the cohort was randomly divided into two datasets—a training/validation dataset (96 (49%) patients) and a testing dataset (100 (51%) patients) for multivariable machine learning.

Summary statistics for all clinical and demographic variables were first calculated. T-tests with unequal variances and Fisher’s exact tests were used to compare quantitative and qualitative variables, respectively, between the training/validation and testing datasets.

Pearson’s correlations were used to examine the correlations between the CT attenuation of the wrist/forearm bones and to evaluate the correlations between CT attenuation of the wrist/forearm bones (distal third of the radius and ulna, proximal third of the first through fifth metacarpals, scaphoid, lunate, triquetrum, pisiform, trapezium, trapezoid, capitate, and hamate) stratified by gender in the entire dataset. Hierarchical cluster analysis was used to cluster the correlations between the CT attenuations of the wrist/forearm bones.

Pearson’s correlation coefficients were also used to evaluate the correlations between the CT attenuations of each of the wrist/forearm bones and DEXA measurements (L1–L4 BMD, L1–L4 BMD T-score, L1–L4 TBS, total hip BMD, total hip BMD T-score, femoral neck BMD, and femoral neck BMD T-score) in the entire dataset.

ROC curves were used to identify the optimal CT attenuation threshold for each bone to evaluate the predictive performance of each bone regarding predicting osteoporosis and osteopenia/osteoporosis in the training/validation dataset. We then evaluated these optimized thresholds in the test dataset.

Since we evaluated the predictive properties of several different bones in the wrist/forearm to predict osteoporosis and osteopenia/osteoporosis, we used several machine learning methods to select the best combination of bones and clinical factors (age, gender, height, weight, BMI) that could be used to categorize a patient as (i) osteoporotic, (ii) osteopenic/osteoporotic using the WHO guidelines, (iii) femoral neck BMD T-score ≤ −2.5, and (iv) femoral neck BMD T-score < −1. We evaluated the femoral neck BMD T-scores because these scores are thought to be less affected by degenerative changes in the lumbar spine and hip.

A support vector machine (SVM) is a supervised learning model that is often used for pattern recognition, classification, and regression analysis [24]. C-classification with three different kernels (linear, radial basis function (RBF), and sigmoid) [24] tuned with 10-fold cross-validation was utilized.

(1) $$\mathrm{Linear}\;\mathrm{kernel},\;K\left(x,y\right)=x.y$$

(2) $$\mathrm{Radial}\;\mathrm{basis}\;\mathrm{function},\;K\left(x,y\right)=e^{\left({\left|\left|x-y\right|\right|}^{2}\right)/2\sigma^{2}}$$

(3) $$\mathrm{Sigmoid}\;\mathrm{kernel},\;K\left(x,y\right)=\tan h\left(\upsilon \left(x.y\right)+c\right)$$

The tuning for each SVM was performed over epsilon ranges from 0 to 1 with 0.1 increments, with cost values ranging from 1 to 6 with increments of 1 using 10-fold cross-validation.

A random forest classifier is an ensemble learning method for classification based on constructing a multitude of decision trees during training [25]. The random forest model was fit and tuned with the number of variables tried at each step starting at 6, a step factor of 1.5, and 10,000 trees used during the tuning step.

Each of the four machine learning models (linear kernel SVM, RBF kernel SVM, sigmoid kernel SVM, random forest classifier) were used to each categorize patients as (i) osteoporotic using WHO guidelines, (ii) osteopenic/osteoporotic using WHO guidelines, (iii) femoral neck BMD T-score ≤ −2.5, and (iv) femoral neck BMD T-score < −1. The optimal models tuned in the training/validation dataset were retained for each analysis. We used these models to predict (i) osteoporotic using WHO guidelines, (ii) osteopenic/osteoporotic using WHO guidelines, (iii) femoral neck BMD T-score ≤ −2.5, and (iv) femoral neck BMD T-score < −1.

All test statistics were two-sided and *p*-values < 0.05 were considered statistically significant. We compared the ROC curves using DeLong’s test [26] and compared the machine learning models to the CT attenuation of the radius 33% since this region is used to determine osteoporosis and osteopenia on DEXA studies of the forearm/wrist. Statistics were performed using the *pROC*, *e1071*, and *RandomForest* packages in *Rv4.1.2* statistical software.

## 3. Results

The entire dataset comprised 196 individuals with a median age of 65.0 years (range 50.0–88.0 years), 173 (88.3%) were females, 54 (27.6%) were osteoporotic, 116 (59.2%) were osteopenic, and the other 26 (13.3%) were normal. There were no statistically significant differences between the variables in the training/validation dataset and the test dataset (Table 1).

**Figure 2:**
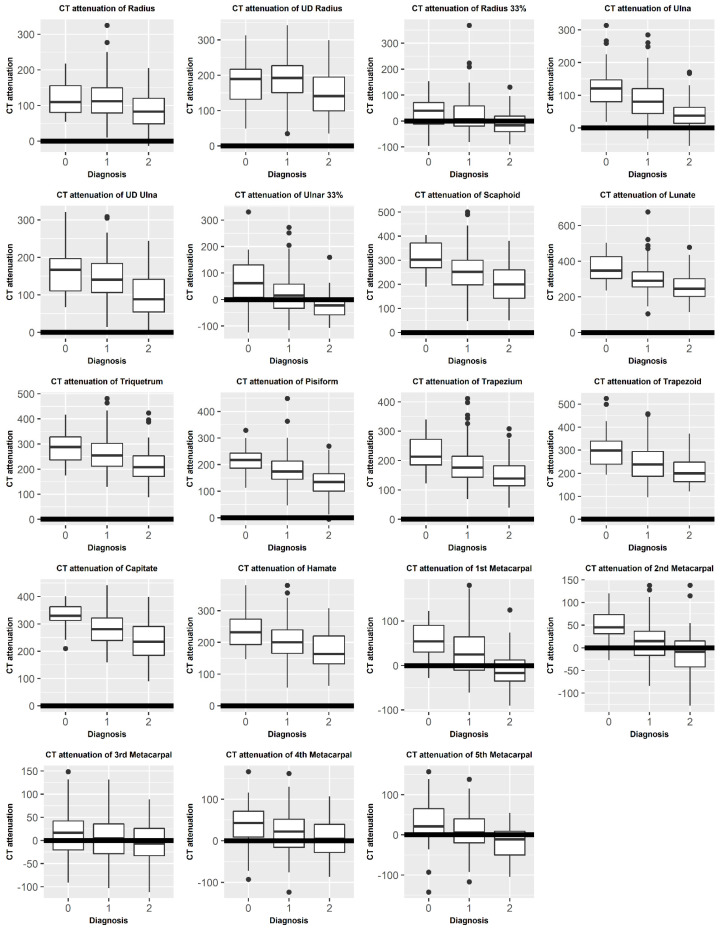
Box and whisker plots of the CT attenuation of each bone of the wrist and forearm by diagnosis: 2—osteoporosis, 1—osteopenia, and 0—normal.

Figure 2 compares the mean CT attenuation of each bone between diagnoses (osteoporosis, osteopenia, and normal).

There were positive correlations between the CT attenuation of the wrist/forearm bones. In women, the strongest CT attenuation correlations were between the CT attenuations of the ulna and ulna UD (r = 0.74, *p* < 0.001), ulna and ulna 33% (r = 0.83, *p* < 0.001), radius and radius UD (r = 0.74, *p* < 0.001), capitate and trapezoid (r = 0.74, *p* < 0.001), hamate and capitate (r = 0.81, *p* < 0.001), and the hamate and trapezoid (r = 0.81, *p* < 0.001). In men, the strongest CT attenuation correlations were between the CT attenuations of radius and the radius UD (r = 0.82, *p* < 0.001), radius and radius 33% (r = 0.79, *p* < 0.001), ulna and ulna UD (r = 0.84, *p* < 0.001), ulna and ulna 33% (r = 0.84, *p* < 0.001), scaphoid and lunate (r = 0.84, *p* < 0.001), triquetrum (r = 0.81, *p* < 0.001), pisiform (r = 0.85, *p* < 0.001), capitate (r = 0.80, *p* < 0.001), lunate and pisiform (r = 0.81, *p* < 0.001), lunate and trapezium (r = 0.80, *p* < 0.001), and trapezium and capitate (r = 0.80, *p* < 0.001) (Table 2).

**Figure 3:**
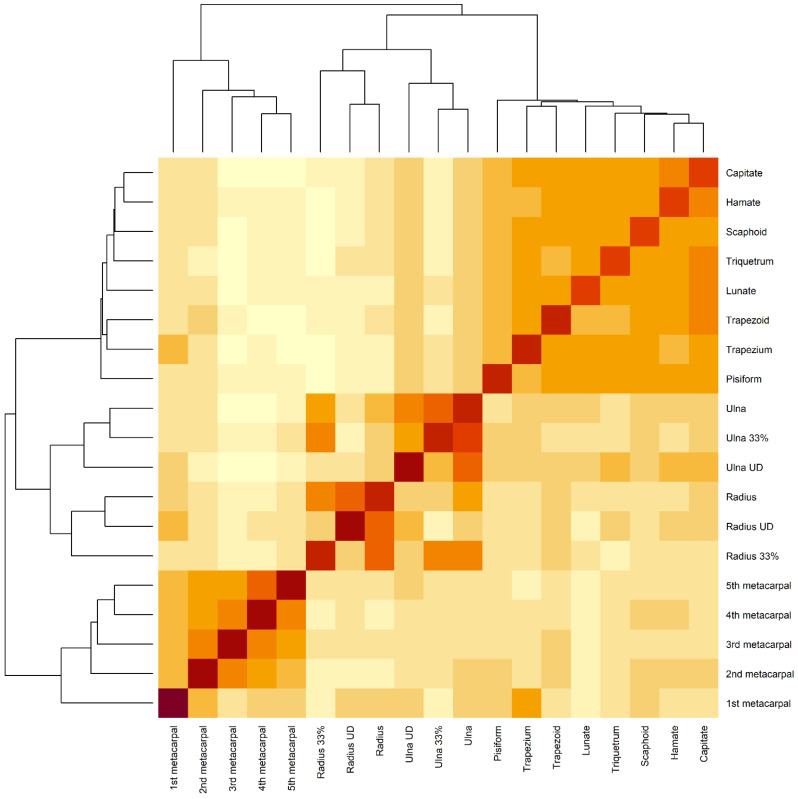
Hierarchical cluster analysis of the correlations between the CT attenuation of each bone of the wrist and forearm. Radius—distal third of the radius; radius UD—ultradistal radius (radius epiphysis and metaphysis); radius 33%—distal third of the radial shaft; ulna—distal third of the ulna; ulna UD—distal ulna (ulnar epiphysis and metaphysis); ulna 33%—distal third of the ulnar shaft; 1st metacarpal—proximal third of the first metacarpal; 2nd metacarpal—proximal third of the second metacarpal; 3rd metacarpal—proximal third of the third metacarpal; 4th metacarpal—proximal third of the fourth metacarpal; 5th metacarpal—proximal third of the fifth metacarpal.

Figure 3 shows that the radial and ulnar measurements clustered together and were highly correlated, whereas the metacarpal measurements clustered together and were highly correlated. Similarly, the carpal measurements clustered together and were highly correlated.

Similarly, we noted strong positive correlations between the CT attenuation of the wrist/forearm bones and DEXA measurements. The strongest correlations between CT attenuations and DEXA L1–L4 BMD T-scores were with the CT attenuation of the first metacarpal (r = 0.26, *p* < 0.001), trapezium (r = 0.26, *p* < 0.001), and scaphoid (r = 0.32, *p* < 0.001) (Table 3).

The strongest correlations between the femoral neck BMD T-scores and the CT attenuations of the wrist/forearm bones were with the CT attenuations of the ulna (r = 0.40, *p* < 0.001), scaphoid (r = 0.47, *p* < 0.001), lunate (r = 0.40, *p* < 0.001), pisiform (r = 0.44, *p* < 0.001), trapezoid (r = 0.40, *p* < 0.001), and capitate (r = 46, *p* < 0.001). We also found that the strongest correlations between the total hip BMD T-score and CT attenuation of the wrist/forearm bones were with the CT attenuation of the scaphoid (r = 0.48, *p* < 0.001), pisiform (r = 0.43, *p* < 0.001), and first metacarpal (r = 0.40, *p* < 0.001). The strongest correlations with the L1–L4 BMD TBS were with the CT attenuation of the scaphoid (r = 0.24, *p* < 0.001), lunate (r = 0.23, *p* < 0.001), trapezium (r = 0.23, *p* < 0.001), and first metacarpal (r = 0.23, *p* < 0.001).

The optimal CT attenuation thresholds for each bone and the predictive performance of these optimal thresholds regarding predicting (i) osteoporosis, (ii) osteopenia/osteoporosis, (iii) femoral neck BMD T-score ≤ −2.5, and (iv) and femoral neck BMD T-score <−1 are shown in Table 4.

### 3.1. Predicting Osteoporosis

#### 3.1.1. Training/Validation Dataset

We found that the CT attenuation of each bone was a significant predictor of osteoporosis in the training/validation dataset (Supplementary Figure S1). The highest AUC was for the hamate (optimal CT threshold 170.166 Hounsfield units (HU), AUC = 0.769), capitate (optimal CT threshold 248.039 HU, AUC = 0.763), and first metacarpal (optimal CT threshold −7.772 HU, AUC = 0.752). The radius 33% had an AUC of 0.705 in the training/validation dataset. The linear kernel SVM (AUC = 0.894) and radial basis function (RBF) kernel SVM (AUC = 0.987) had the highest AUCs of the machine learning models in the training/validation dataset.

#### 3.1.2. Test Dataset

The performances of the CT attenuation thresholds obtained from the training/validation dataset were evaluated in the test dataset. The hamate (AUC = 0.393), capitate (AUC = 0.636), first metacarpal (AUC = 0.639), and radius 33% (AUC = 0.576) showed slightly lower predictive abilities in the test dataset compared to the training dataset. The RBF kernel SVM (AUC = 0.818) had a higher AUC than any of the CT attenuation thresholds for each bone. The RBF kernel SVM was better than the radius 33% model (*p* = 0.020).

### 3.2. Predicting Osteopenia/Osteoporosis

#### 3.2.1. Training/Validation Dataset

When predicting osteopenia/osteoporosis in the training/validation dataset, we found that the CT attenuation of each bone was predictive of osteoporosis/osteopenia (Supplementary Figure S2). The first metacarpal (optimal CT threshold 27.779 HU, AUC = 0.823), scaphoid (optimal CT attenuation threshold 250.749 HU, AUC = 0.773), and lunate (optimal CT attenuation threshold 258.091 HU, AUC = 0.768) were the most predictive, whereas the radius UD (AUC = 0.528), third metacarpal (AUC = 0.529), and fourth metacarpal (AUC = 0.579) were the least predictive bones. The radius 33% had an AUC of 0.716 regarding predicting osteopenia/osteoporosis in the training/validation dataset. The RBF kernel SVM (AUC = 0.969) had the highest AUC of all the machine learning methods investigated regarding predicting osteopenia/osteoporosis in the training/validation dataset.

#### 3.2.2. Test Dataset

In the test dataset, we found that the CT attenuations thresholds obtained from the training/validation dataset for the first metacarpal (AUC = 0.445), scaphoid (AUC = 0.651), and lunate (AUC = 0.433) were less predictive in the test dataset. The radius 33% CT attenuation threshold had an AUC of 0.563 in the test dataset. However, the RBF kernel SVM (AUC = 0.805) had the highest AUC and accuracy in the test dataset. The RBF kernel SVM (*p* = 0.068) was not significantly better than the radius 33% in the test dataset.

### 3.3. Predicting Femoral Neck BMD T-Score ≤ −2.5

#### 3.3.1. Training/Validation Dataset

When predicting femoral neck BMD T-score ≤ −2.5, we found that the CT attenuation of each of the bones studied was predictive (Supplementary Figure S3). The fifth metacarpal (optimal CT attenuation threshold 24.690 HU, AUC = 0.737), pisiform (optimal CT attenuation threshold 121.626 HU, AUC = 0.736), and first metacarpal (optimal CT attenuation threshold 0.530 HU, AUC = 0.710) were the best bones for predicting femoral neck BMD T-score ≤ −2.5 in the training/validation dataset, whereas the CT attenuation of the radius (AUC = 0.569), ulna UD (AUC = 0.581), and lunate (AUC = 0.600) were the bones that were the worst predictors of femoral neck BMD T-score ≤ −2.5. The RBF kernel SVM (AUC = 0.997) had the highest AUC in the training/validation dataset.

#### 3.3.2. Test Dataset

In the test dataset, we found that the CT attenuation thresholds obtained from the training/validation dataset for the fifth metacarpal (AUC = 0.398), pisiform (AUC = 0.415), first metacarpal (AUC = 0.618), and radius 33% (AUC = 0.426) were less predictive. The RBF kernel SVM had an AUC of 0.770 regarding identifying patients with a femoral neck BMD T-score ≤ −2.5. The RBF kernel SVM (AUC = 0.770, *p* < 0.001) model was better than the model using the radius 33% CT attenuation threshold.

### 3.4. Predicting Femoral Neck BMD T-Score < −1

#### 3.4.1. Training/Validation Dataset

We found that a CT attenuation threshold of the trapezium (optimal CT attenuation threshold 165.624 HU, AUC = 0.722) had the best AUC to predict femoral neck BMD T-score <−1 in the training/validation dataset (Supplementary Figure S4). The scaphoid (optimal CT attenuation threshold 229.799 HU, AUC = 0.719) and pisiform (optimal CT attenuation threshold 221.709 HU, AUC = 0.714) were the next best predictors of having a femoral neck BMD T-score < −1. The radius 33% (AUC = 0.605), radius (AUC = 0.603), radius UD (AUC = 0.558), and hamate (AUC = 0.584) were the worst predictors of having a femoral neck BMD < −1 in the training/validation dataset. The RBF kernel SVM (AUC = 0.987) had the highest AUC of all the multivariable machine learning models regarding predicting femoral neck BMD T-score < −1 in the training/validation dataset.

#### 3.4.2. Test Dataset

In the test dataset, we found that the CT attenuations thresholds obtained from the training/validation dataset for the scaphoid (AUC = 0.736), trapezium (AUC = 0.714), pisiform (AUC = 0.437), and radius 33% (AUC = 0.423) were less predictive in the test dataset. The RBF kernel SVM (AUC = 0.818, *p* < 0.001) had a significantly higher AUC than radius 33% (AUC = 0.423) in the test dataset.

## 4. Discussion

We found strong positive correlations between the CT attenuation of the wrist/forearm bones. There were stronger correlations between the CT attenuations of the radius and ulna measurements, stronger correlations between the carpal CT attenuations, and stronger correlations between the metacarpal CT attenuations. The CT attenuations of the wrist/forearm bones were largely positively correlated with the DEXA measurements. The RBF kernel SVM was best for predicting osteoporosis with a higher AUC (AUC = 0.818) than other models and statistically better than radius 33%. The RBF kernel SVM also had the highest AUC (AUC = 0.805) regarding predicting patients with osteoporosis/osteopenia. We found that the RBF kernel SVM was best for predicting a femoral neck BMD T-score ≤ −2.5 and was statistically better than the CT attenuation of radius 33%. The data also showed that the RBF kernel SVM was the best for predicting a femoral neck BMD T-score < −1 and statistically better than the CT attenuation of radius 33%.

These results have significant clinical implications. We showed that opportunistic screening for osteoporosis and osteopenia can be performed using routine CT scans of the wrist/forearm obtained for clinical care. We also showed that the accuracy of a machine learning model using the CT attenuation of multiple bones and clinical/demographic variables exceeded that of a single bone. We provided CT attenuation thresholds for each wrist/forearm bone that could be used to identify patients who should go on to get screened for osteoporosis or osteopenia/osteoporosis with a formal DEXA study. This has the potential to identify patients earlier in their course of BMD loss and get patients to clinical care earlier.

The radius transmits the majority of the force from the wrist to the elbow and is therefore often fractured when individuals with diminished BMD fall on an outstretched hand [6,7,27]. Forearm fractures, in particular, distal radius fractures, are often frailty fractures related to diminished BMD. Although the radius is often evaluated on DEXA studies for the prediction of osteopenia/osteoporosis and osteoporosis, we found that the CT attenuation of the radius was not the single best bone for predicting osteopenia/osteoporosis and osteoporosis in the analysis. Bone homeostasis is regulated by the activities of three cell types, namely, the osteoclasts, osteocytes, and osteoblasts, and is generally kept in dynamic equilibrium to maintain bone mass [28]. Our data suggested that all bones may be differentially affected when there is diminished bone mass because of the lack of perfect correlation between the CT attenuation of bones and the likely BMD of each bone [29]. Each bone also likely has a different trabecular bone structure in order to best suit its mechanical/structural demands, and this may affect the mean CT attenuation between bones.

The CT attenuation of the hamate had the best performance to differentiate patients with osteoporosis from those without osteoporosis. The patients in this study were aged 50 years or older, and likely had degenerative changes of the wrist. Degenerative changes of the carpus often affect the scaphoid, trapezium, and trapezoid (triscaphe and first carpometacarpal joint degenerative changes), scapholunate, and the pisotriquetral articulations. These degenerative changes are manifested by increased sclerosis and eburnation, and this increased sclerosis likely affects the CT attenuation of each bone. We hypothesized that the hamate was least likely to be affected by degenerative changes and this was likely the reason it had the best performance. One strength of the multivariable SVM and random forest models was that these models utilized the CT attenuation of all the bones and, therefore, had better performance than the CT attenuation of any individual bone.

A prior study noted that the second metacarpal cortical index can be used for opportunistic screening for osteoporosis [22]. We showed that the CT attenuation of the second metacarpal is predictive of osteoporosis and osteopenia/osteoporosis. We showed that the CT attenuation of the other metacarpals was also predictive of osteoporosis and osteopenia/osteoporosis. However, the CT attenuation of the metacarpals was less accurate than the multivariable machine learning models for the diagnosis of osteoporosis and osteopenia/osteoporosis.

One limitation of this study was the small sample size. We found that most patients with CT scans of the wrist/forearm at our institution did not have concurrent DEXA studies, which suggests that there is under-screening/inadequate screening for osteopenia and osteoporosis. Our method may help to identify patients who had a CT scan of the wrist/forearm who should go on to have a formal DEXA study to screen for osteopenia and osteoporosis in the near future. Patients all had their CT scans of the wrist/forearm performed using Siemens scanners, which is a limitation; however, a recently published article suggests that there is minimal bias in CT attenuation measurements between CT manufacturers [30]. This study was a retrospective study at a single multi-center tertiary care academic institution. Another limitation is that most patients were white; therefore, it is unclear how our results will port to other races/ethnicities. While we showed methods regarding how to categorize patients as osteoporotic or osteopenic/osteoporotic using the CT attenuation of the wrist/forearm bones based on DEXA studies, further work is required to evaluate how well these measurements predict future frailty fractures.

## 5. Conclusions

In summary, opportunistic screening for osteoporosis and osteopenia/osteoporosis could be performed using the CT attenuation of each of the bones from CT scans of the wrist/forearm. We used machine learning to show that using the CT attenuation of multiple bones was more accurate than using the CT attenuation of a single bone. DEXA scans currently evaluate only the lumbar spine and the hips to assess global bone mineral density. CT attenuation data from routine CT scans of the wrist and forearm can be used to identify patients at risk for osteoporosis who should go on to have formal screening for osteoporosis using DEXA scans.

## Figures and Tables

**Table 1:** Comparison between training/validation and test datasets.

Variable	All (N = 196)	Training/ Validation Dataset (N = 96)	Test Dataset (N = 100)	*p*-Value
Age	64.9 (8.7)	64.5 (8.8)	65.2 (8.7)	0.593
Race/ethnicity				0.248
American Indian/Alaskan native	1 (0.5%)	1 (1.0%)	0 (0.0%)	
Asian	2 (1.0%)	0 (0.0%)	2 (2.0%)	
Black/African-American	1 (0.5%)	0 (0.0%)	1 (1.0%)	
Hispanic	2 (1.0%)	0 (0.0%)	2 (2.0%)	
Other	1 (0.5%)	0 (0.0%)	1 (1.0%)	
White	189 (96.4%)	95 (99.0%)	94 (94.0%)	
Gender, male	23 (11.7%)	12 (12.5%)	11 (11.0%)	0.826
Height (m)	1.65 (0.08)	1.65 (0.08)	1.64 (0.08)	0.47
Weight (kg)	76.2 (19.2)	76.5 (19.1)	75.8 (19.4)	0.8
BMI (kg/m^2^)	27.9 (6.4)	27.9 (6.4)	27.9 (6.4)	0.991
Diagnosis				1
Osteoporosis	54 (27.6%)	26 (27.1%)	28 (28.0%)	
Osteopenia	116 (59.2%)	57 (59.4%)	59 (59.0%)	
Normal	26 (13.3%)	13 (13.5%)	13 (13.0%)	

BMI—body mass index; osteoporosis—minimum BMD T-score ≤ −2.5; osteopenia—−2.5 < minimum BMD T-score < −1; normal—minimum BMD T-score ≥ −1; ***—*p*-value < 1 × 10^−3^; **—1 × 10^−3^ < *p*-value < 1 × 10^−2^; *—1 × 10^−2^ < *p*-value < 5 × 10^−2^.

**Table 2:** Correlations between the CT attenuations at different bony sites in males and females.

Males Top Diagonal	Radius	Radius UD	Radius 33%	Ulna	Ulna UD	Ulna 33%	Scaphoid	Lunate	Triquetrum	Pisiform	Trapezium	Trapezoid	Capitate	Hamate	1 MC	2 MC	3 MC	4 MC	5 MC
Females bottom diagonal																			
Radius	-	0.82 ***	0.79 ***	0.55 **	0.46 *	0.32	0.37	0.36	0.31	0.34	0.40	0.56 **	0.50 *	0.30	0.46 *	0.12	0.08	−0.19	0.05
Radius UD	0.74 ***	-	0.45 *	0.51 *	0.55 **	0.22	0.54 **	0.52 *	0.56 **	0.47 *	0.57 **	0.61 **	0.59 **	0.39	0.64 **	0.24	0.27	0.03	0.12
Radius 33%	0.73 ***	0.40 ***	-	0.57 **	0.35	0.56 **	0.25	0.18	0.04	0.27	0.24	0.41	0.31	0.21	0.30	0.00	−0.09	−0.03	0.04
Ulna	0.61 ***	0.39 ***	0.64 ***	-	0.84 ***	0.84 ***	0.62 **	0.57 **	0.37	0.65 ***	0.62 **	0.62 **	0.65 ***	0.49 *	0.43 *	0.32	0.09	0.27	0.10
Ulna UD	0.46 ***	0.43 ***	0.35 ***	0.74 ***	-	0.62 **	0.64 **	0.70 ***	0.46 *	0.62 **	0.67 ***	0.68 ***	0.67 ***	0.39	0.57 **	0.32	0.24	0.30	0.24
Ulna 33%	0.46 ***	0.24 **	0.69 ***	0.83 ***	0.52 ***	-	0.54 **	0.43 *	0.22	0.59 **	0.50 *	0.36	0.41	0.42 *	0.35	0.33	0.06	0.49 *	0.23
Scaphoid	0.38 ***	0.28 ***	0.25 ***	0.42 ***	0.45 ***	0.30 ***	-	0.84 ***	0.81 ***	0.85 ***	0.78 ***	0.71 ***	0.80 ***	0.66 ***	0.46 *	0.55 **	0.45 *	0.32	0.27
Lunate	0.27 ***	0.15 *	0.23 **	0.42 ***	0.42 ***	0.30 ***	0.60 ***	-	0.76 ***	0.81 ***	0.80 ***	0.75 ***	0.78 ***	0.58 **	0.59 **	0.55 **	0.50 *	0.33	0.42 *
Triquetrum	0.37 ***	0.33 ***	0.22 **	0.41 ***	0.51 ***	0.26 ***	0.66 ***	0.64 ***	-	0.69 ***	0.71 ***	0.59 **	0.77 ***	0.61 **	0.50 *	0.59 **	0.55 **	0.29	0.45 *
Pisiform	0.28 ***	0.25 ***	0.20 ***	0.37 ***	0.46 ***	0.34 ***	0.59 ***	0.55 ***	0.59 ***	-	0.74 ***	0.72 ***	0.69 ***	0.56 **	0.54 **	0.51 *	0.31	0.37	0.33
Trapezium	0.31 ***	0.23**	0.19*	0.38 ***	0.41 ***	0.31 ***	0.66 ***	0.57 ***	0.61 ***	0.51 ***	-	0.74 ***	0.80 ***	0.53 *	0.71 ***	0.59 **	0.68 ***	0.51 *	0.41
Trapezoid	0.40 ***	0.29 ***	0.26 ***	0.40 ***	0.38 ***	0.24 **	0.66 ***	0.57 ***	0.58 ***	0.56 ***	0.59 ***	-	0.74 ***	0.38	0.53 **	0.50 *	0.39	0.14	0.19
Capitate	0.39 ***	0.31 ***	0.24 **	0.45 ***	0.47 ***	0.33 ***	0.71 ***	0.72 ***	0.73 ***	0.62 ***	0.66 ***	0.74 ***	-	0.62 **	0.55 **	0.41	0.45 *	0.16	0.32
Hamate	0.38 ***	0.34 ***	0.25 ***	0.46 ***	0.50 ***	0.31 ***	0.66 ***	0.65 ***	0.69 ***	0.62 ***	0.62 ***	0.69 ***	0.81 ***	-	0.28	0.29	0.29	0.18	0.17
1 MC	0.41 ***	0.39 ***	0.27 ***	0.39 ***	0.38 ***	0.31 ***	0.44 ***	0.24 **	0.34 ***	0.38 ***	0.48 ***	0.35 ***	0.35 ***	0.38 ***	-	0.46 *	0.52 *	0.51 *	0.64 **
2 MC	0.32 ***	0.27 ***	0.25 ***	0.37 ***	0.29 ***	0.33 ***	0.35 ***	0.22 **	0.22 **	0.34 ***	0.31 ***	0.42 ***	0.37 ***	0.37 ***	0.48 ***	-	0.72 ***	0.63 **	0.68 ***
3 MC	0.23 ***	0.20 **	0.26 ***	0.22 **	0.18 *	0.23 **	0.24 **	0.06	0.13	0.24 ***	0.15 *	0.28 ***	0.20 **	0.26 ***	0.36 ***	0.63 ***	-	0.60 **	0.68 ***
4 MC	0.27 ***	0.32 ***	0.23 **	0.19 *	0.22 **	0.19*	0.30 ***	0.15	0.24**	0.21**	0.19*	0.25 ***	0.24**	0.34 ***	0.45 ***	0.55 ***	0.68 ***	-	0.65 ***
5 MC	0.34 ***	0.31 ***	0.30 ***	0.28 ***	0.28 ***	0.26 ***	0.24 **	0.14	0.22 **	0.27 ***	0.14	0.20 **	0.20 **	0.26 ***	0.37 ***	0.49 ***	0.54 ***	0.70 ***	-

Radius—distal third of the radius; radius UD—ultradistal radius (radius epiphysis/metaphysis); radius 33%—distal third of the radial shaft; ulna—distal third of the ulna; ulna UD—distal ulna (ulnar epiphysis/metaphysis); ulna 33%—distal third of the ulnar shaft; 1 MC—proximal third of the first metacarpal; 2 MC—proximal third of the second metacarpal; 3 MC—proximal third of the third metacarpal; 4 MC—proximal third of the fourth metacarpal; 5 MC—proximal third of the fifth metacarpal; ***—*p*-value < 1 × 10^−3^; **—1 × 10^−3^ < *p*-value < 1 × 10^−2^; *—1 × 10^−2^ < *p*-value < 5 × 10^−2^.

**Table 3:** Correlations between the CT attenuation at different bony sites and DEXA measurements.

All	L1–L4 BMD	L1–L4 BMD T-Score	L1–L4 TBS (N = 133)	Femoral Neck BMD	Femoral Neck BMD T-Score	Total Hip BMD	Total Hip BMD T-Score
Radius	0.04	0.03	0.12	0.26 ***	0.24 ***	0.29 ***	0.26 ***
Radius UD	0.00	−0.02	0.08	0.23 ***	0.17 *	0.18 *	0.14 *
Radius 33%	0.11	0.13	0.11	0.23 **	0.25 ***	0.31 ***	0.29 ***
Ulna	0.23 **	0.24 ***	0.18 *	0.40 ***	0.40 ***	0.43 ***	0.38 ***
Ulna UD	0.27 ***	0.17*	0.22*	0.40 ***	0.39 ***	0.42 ***	0.38 ***
Ulna 33%	0.24 ***	0.24 ***	0.19 *	0.34 ***	0.37 ***	0.37 ***	0.35 ***
Scaphoid	0.26 ***	0.32 ***	0.24 **	0.39 ***	0.47 ***	0.48 ***	0.48 ***
Lunate	0.22 **	0.23 **	0.23 **	0.36 ***	0.40 ***	0.40 ***	0.38 ***
Triquetrum	0.14 *	0.17 *	0.18 *	0.33 ***	0.38 ***	0.40 ***	0.39 ***
Pisiform	0.18 *	0.25 ***	0.17 *	0.40 ***	0.44 ***	0.47 ***	0.43 ***
Trapezium	0.28 ***	0.26 ***	0.23 **	0.32 ***	0.38 ***	0.40 ***	0.37 ***
Trapezoid	0.15 *	0.18 *	0.15	0.35 ***	0.40 ***	0.38 ***	0.34 ***
Capitate	0.19 **	0.24 ***	0.21 *	0.39 ***	0.46 ***	0.43 ***	0.42 ***
Hamate	0.21 **	0.22 **	0.14	0.30 ***	0.35 ***	0.35 ***	0.34 ***
1 MC	0.24 ***	0.26 ***	0.17	0.36 ***	0.38 ***	0.43 ***	0.40 ***
2 MC	0.24 ***	0.19 **	0.23 **	0.23 **	0.31 ***	0.37 ***	0.34 ***
3 MC	0.18 *	0.04	0.21 *	0.15 *	0.17 *	0.21 **	0.19 **
4 MC	0.22 **	0.10	0.15	0.17 *	0.23 **	0.27 ***	0.27 ***
5 MC	0.27 ***	0.16 *	0.16	0.29 ***	0.32 ***	0.38 ***	0.37 ***

Radius—distal third of the radius; radius UD—ultradistal radius (radius epiphysis/metaphysis); radius 33%—distal third of the radial shaft; ulna—distal third of the ulna; ulna UD—distal ulna (ulnar epiphysis/metaphysis); ulna 33%—distal third of the ulnar shaft; 1 MC—proximal third of the first metacarpal; 2 MC—proximal third of the second metacarpal; 3 MC—proximal third of the third metacarpal; 4 MC—proximal third of the fourth metacarpal; 5 MC—proximal third of the fifth metacarpal; ***—*p*-value < 1 × 10^−3^; **—1 × 10^−3^ < *p*-value < 1 × 10^−2^; *—1 × 10^−2^ < *p*-value < 5 × 10^−2^.

**Table 4:** Performance of the CT attenuation of each bone and multivariable machine learning models to predict osteoporosis and osteopenia/osteoporosis.

			Test Dataset					
**Osteoporosis**	**Training/** **Validation** **Dataset CT** **Attenuation Threshold**	**AUC**	**Sensitivity**	**Specificity**	**AUC**	**Accuracy**	**Positive Predictive Value (PPV)**	**Negative Predictive Value (NPV)**
Radius	90.179	0.708	0.500	0.639	0.569	0.600	0.350	0.767
Radius UD	154.998	0.725	0.607	0.625	0.616	0.620	0.386	0.804
Radius 33%	−13.717	0.705	0.500	0.653	0.576	0.610	0.359	0.770
Ulna	67.121	0.719	0.750	0.667	0.708	0.690	0.467	0.873
Ulna UD	98.446	0.732	0.500	0.806	0.653	0.720	0.500	0.806
Ulna 33%	3.872	0.669	0.750	0.611	0.681	0.650	0.429	0.863
Scaphoid	247.592	0.763	0.571	0.583	0.577	0.580	0.348	0.778
Lunate	248.387	0.762	0.00	1.00	0.365	0.720	-	0.720
Triquetrum	207.882	0.730	0.00	1.00	0.390	0.720	-	0.720
Pisiform	162.298	0.753	0.714	0.653	0.684	0.670	0.444	0.855
Trapezium	141.824	0.734	0.00	1.00	0.383	0.720	-	0.720
Trapezoid	231.070	0.699	0.500	0.722	0.611	0.660	0.412	0.788
Capitate	248.039	0.763	0.536	0.736	0.636	0.680	0.441	0.803
Hamate	170.166	0.769	0.00	1.00	0.393	0.720	-	0.720
1 MC	−7.772	0.752	0.500	0.778	0.639	0.700	0.467	0.800
2 MC	16.023	0.686	0.00	1.00	0.415	0.720	-	0.720
3 MC	61.555	0.565	0.00	1.00	0.466	0.720	-	0.720
4 MC	50.837	0.600	0.00	1.00	0.415	0.720	-	0.720
5 MC	−34.860	0.566	0.00	1.00	0.408	0.720	-	0.720
Linear kernel SVM		0.894	0.883	0.435	0.680	0.780	0.840	0.526
Radial basis function kernel SVM		0.987	0.584	0.957	0.818	0.670	0.978	0.407
Sigmoid kernel SVM		0.627	0.844	0.739	0.818	0.820	0.915	0.586
Random Forest classifier		0.502	0.987	0.087	0.537	0.780	0.784	0.667
**Osteopenia/Osteoporosis**	**Training/** **Validation** **Dataset CT** **Attenuation** **Threshold**	**AUC**	**Sensitivity**	**Specificity**	**AUC**	**Accuracy**	**Positive Predictive Value (PPV)**	**Negative Predictive Value (NPV)**
Radius	149.199	0.635	0.262	0.778	0.520	0.329	0.889	0.135
Radius UD	160.496	0.528	0.00	1.00	0.472	0.129	-	0.129
Radius 33%	10.942	0.716	0.459	0.667	0.563	0.486	0.903	0.154
Ulna	117.259	0.736	0.00	1.00	0.432	0.129	-	0.129
Ulna UD	162.088	0.643	0.705	0.556	0.630	0.686	0.915	0.217
Ulna 33%	73.365	0.708	0.00	1.00	0.454	0.129	-	0.129
Scaphoid	250.749	0.773	0.525	0.778	0.651	0.557	0.941	0.194
Lunate	258.091	0.768	0.00	1.00	0.433	0.129	-	0.129
Triquetrum	213.998	0.610	0.00	1.00	0.392	0.129	-	0.129
Pisiform	220.041	0.754	0.00	1.00	0.423	0.129	-	0.129
Trapezium	183.738	0.717	0.00	1.00	0.310	0.129	-	0.129
Trapezoid	269.594	0.726	0.656	0.778	0.717	0.671	0.952	0.250
Capitate	294.058	0.755	0.623	0.889	0.756	0.657	0.974	0.258
Hamate	171.503	0.673	0.00	1.00	0.423	0.129	-	0.129
1 MC	27.779	0.823	0.00	1.00	0.445	0.129	-	0.129
2 MC	30.584	0.752	0.721	0.889	0.805	0.743	0.978	0.320
3 MC	31.197	0.529	0.00	1.00	0.409	0.129	-	0.129
4 MC	55.376	0.579	0.770	0.556	0.663	0.743	0.922	0.263
5 MC	52.112	0.615	0.00	1.00	0.407	0.390	-	0.390
Linear kernel SVM		0.856	0.443	0.889	0.674	0.620	0.871	0.507
Radial basis function kernel SVM		0.969	0.885	0.667	0.805	0.800	0.806	0.788
Sigmoid kernel SVM		0.542	0.607	0.778	0.716	0.670	0.804	0.556
Random Forest classifier		0.511	0.967	0.222	0.595	0.680	0.663	0.818
**Femoral Neck BMD ≤ −2.5**	**Training/** **Validation** **Dataset CT** **Attenuation** **Threshold**	**AUC**	**Sensitivity**	**Specificity**	**AUC**	**Accuracy**	**Positive Predictive Value (PPV)**	**Negative Predictive Value (NPV)**
Radius	132.495	0.569	0.00	1.00	0.394	0.810	-	0.810
Radius UD	184.154	0.618	0.789	0.531	0.660	0.580	0.283	0.915
Radius 33%	20.908	0.625	0.00	1.00	0.426	0.810	-	0.810
Ulna	67.121	0.603	0.789	0.556	0.673	0.600	0.294	0.918
Ulna UD	82.730	0.581	0.526	0.790	0.658	0.740	0.370	0.877
Ulna 33%	35.520	0.621	0.00	1.00	0.375	0.810	-	0.810
Scaphoid	202.916	0.657	0.632	0.679	0.655	0.670	0.316	0.887
Lunate	224.838	0.684	0.526	0.864	0.695	0.800	0.476	0.886
Triquetrum	208.334	0.667	0.632	0.728	0.680	0.710	0.353	0.894
Pisiform	121.626	0.736	0.00	1.00	0.415	0.810	-	0.810
Trapezium	149.597	0.627	0.632	0.691	0.661	0.680	0.324	0.889
Trapezoid	207.953	0.663	0.632	0.679	0.655	0.670	0.316	0.887
Capitate	248.039	0.647	0.737	0.667	0.702	0.680	0.341	0.915
Hamate	185.743	0.600	0.842	0.568	0.705	0.620	0.314	0.939
1 MC	0.530	0.710	0.579	0.642	0.610	0.630	0.275	0.867
2 MC	−7.273	0.681	0.526	0.630	0.578	0.610	0.250	0.850
3 MC	−47.251	0.609	0.895	0.136	0.515	0.280	0.195	0.846
4 MC	−13.146	0.672	0.00	1.00	0.458	0.810	-	0.810
5 MC	24.690	0.737	0.00	1.00	0.398	0.810	-	0.810
Linear kernel SVM		0.915	0.947	0.593	0.795	0.660	0.535	0.980
Radial basis function kernel SVM		0.997	0.579	0.864	0.770	0.810	0.500	0.897
Sigmoid kernel SVM		0.736	0.947	0.531	0.749	0.610	0.321	0.977
Random Forest classifier		0.489	0.421	0.901	0.661	0.810	0.500	0.869
**Femoral Neck BMD < −1**	**Training/** **Validation** **Dataset CT** **Attenuation** **Threshold**	**AUC**	**Sensitivity**	**Specificity**	**AUC**	**Accuracy**	**Positive Predictive Value (PPV)**	**Negative Predictive Value (NPV)**
Radius	130.336	0.603	0.00	1.00	0.415	0.270	-	0.270
Radius UD	163.209	0.558	0.00	1.00	0.492	0.270	-	0.270
Radius 33%	10.942	0.605	0.00	1.00	0.423	0.270	-	0.270
Ulna	94.009	0.647	0.740	0.652	0.696	0.720	0.857	0.486
Ulna UD	185.544	0.684	0.00	1.00	0.363	0.270	-	0.270
Ulna 33%	27.406	0.618	0.727	0.739	0.733	0.730	0.883	0.500
Scaphoid	229.799	0.719	0.558	0.913	0.736	0.660	0.953	0.439
Lunate	268.193	0.707	0.00	1.00	0.331	0.270	-	0.270
Triquetrum	287.366	0.641	0.831	0.565	0.698	0.760	0.836	0.556
Pisiform	221.709	0.714	0.00	1.00	0.437	0.270	-	0.270
Trapezium	165.624	0.722	0.558	0.870	0.714	0.640	0.911	0.418
Trapezoid	236.041	0.693	0.610	0.696	0.653	0.640	0.849	0.404
Capitate	257.499	0.693	0.545	0.870	0.708	0.790	0.842	0.625
Hamate	160.072	0.584	0.00	1.00	0.299	0.270	-	0.270
1 MC	26.390	0.710	0.714	0.609	0.661	0.680	0.825	0.432
2 MC	9.576	0.700	0.623	0.870	0.746	0.680	0.918	0.451
3 MC	54.574	0.491	0.00	1.00	0.424	0.270	-	0.270
4 MC	5.199	0.616	0.00	1.00	0.427	0.270	-	0.270
5 MC	1.294	0.674	0.597	0.696	0.647	0.630	0.846	0.396
Linear kernel SVM		0.895	0.468	0.826	0.678	0.550	0.900	0.317
Radial basis function kernel SVM		0.987	0.584	0.957	0.818	0.670	0.978	0.407
Sigmoid kernel SVM		0.627	0.844	0.739	0.818	0.820	0.915	0.586
Random Forest classifier		0502	0.987	0.043	0.515	0.770	0.776	0.500

Radius—distal third of the radius; Radius UD—ultradistal radius (radius epiphysis/metaphysis); Radius 33%—distal third of the radial shaft; Ulna—distal third of the ulna; Ulna UD—distal ulna (ulnar epiphysis/metaphysis); Ulna 33%—distal third of the ulnar shaft; 1 MC—proximal third of the first metacarpal; 2 MC—proximal third of the second metacarpal; 3 MC—proximal third of the third metacarpal; 4 MC—proximal third of the fourth metacarpal; 5 MC—proximal third of the fifth metacarpal; -—Undefined.

## Data Availability

Data available upon request from the corresponding author and IRB approval.

## Supplementary Materials

The following supporting information can be downloaded at: https://www.mdpi.com/2075-4418/12/3/691/s1, Figure S1: Performance of the CT attenuation of each bone and multivariable machine learning models to predict osteoporosis; Figure S2: Performance of the CT attenuation of each bone and multivariable machine learning models to predict osteopenia/osteoporosis; Figure S3: Performance of the CT attenuation of each bone and multivariable machine learning models to predict femoral neck BMD T-score ≤ −2.5; Figure S4: Performance of the CT attenuation of each bone and multivariable machine learning models to predict femoral neck BMD T-score < −1.
