# Correction: Sebro, R.; De la Garza-Ramos, C. Machine Learning for Opportunistic Screening for Osteoporosis from CT Scans of the Wrist and Forearm. *Diagnostics* 2022, *12*, 691

**DOI:** 10.3390/diagnostics12112635

**Published:** 2022-10-31

**Authors:** Ronnie Sebro, Cynthia De la Garza-Ramos

**Affiliations:** 1 Mayo Clinic Florida, Department of Radiology, Jacksonville, FL 32224, USA; 2 Center for Augmented Intelligence, Mayo Clinic Florida, Department of Radiology, Jacksonville, FL 32224, USA

In the original publication [1], there was an error in Table 4 as published. The positive predicted value was listed as 0, when it is undefined (“-”). The corrected Table 4 appears below. The authors state that the scientific conclusions are unaffected. This correction was approved by the Academic Editor. The original publication has also been updated.

## Figures and Tables

**Table 4:** Performance of the CT attenuation of each bone and multivariable machine learning models to predict osteoporosis and osteopenia/osteoporosis.

			Test Dataset					
Osteoporosis	Training/ Validation Dataset CT Attenuation Threshold	AUC	Sensitivity	Specificity	AUC	Accuracy	Positive Predictive Value (PPV)	Negative Predictive Value (NPV)
Radius	90.179	0.708	0.500	0.639	0.569	0.600	0.350	0.767
Radius UD	154.998	0.725	0.607	0.625	0.616	0.620	0.386	0.804
Radius 33%	−13.717	0.705	0.500	0.653	0.576	0.610	0.359	0.770
Ulna	67.121	0.719	0.750	0.667	0.708	0.690	0.467	0.873
Ulna UD	98.446	0.732	0.500	0.806	0.653	0.720	0.500	0.806
Ulna 33%	3.872	0.669	0.750	0.611	0.681	0.650	0.429	0.863
Scaphoid	247.592	0.763	0.571	0.583	0.577	0.580	0.348	0.778
Lunate	248.387	0.762	0.00	1.00	0.365	0.720	-	0.720
Triquetrum	207.882	0.730	0.00	1.00	0.390	0.720	-	0.720
Pisiform	162.298	0.753	0.714	0.653	0.684	0.670	0.444	0.855
Trapezium	141.824	0.734	0.00	1.00	0.383	0.720	-	0.720
Trapezoid	231.070	0.699	0.500	0.722	0.611	0.660	0.412	0.788
Capitate	248.039	0.763	0.536	0.736	0.636	0.680	0.441	0.803
Hamate	170.166	0.769	0.00	1.00	0.393	0.720	-	0.720
1 MC	−7.772	0.752	0.500	0.778	0.639	0.700	0.467	0.800
2 MC	16.023	0.686	0.00	1.00	0.415	0.720	-	0.720
3 MC	61.555	0.565	0.00	1.00	0.466	0.720	-	0.720
4 MC	50.837	0.600	0.00	1.00	0.415	0.720	-	0.720
5 MC	−34.860	0.566	0.00	1.00	0.408	0.720	-	0.720
Linear kernel SVM		0.894	0.883	0.435	0.680	0.780	0.840	0.526
Radial basis function kernel SVM		0.987	0.584	0.957	0.818	0.670	0.978	0.407
Sigmoid kernel SVM		0.627	0.844	0.739	0.818	0.820	0.915	0.586
Random Forest classifier		0.502	0.987	0.087	0.537	0.780	0.784	0.667
**Osteopenia/Osteoporosis**	**Training/** **Validation** **Dataset CT Attenuation Threshold**	**AUC**	**Sensitivity**	**Specificity**	**AUC**	**Accuracy**	**Positive Predictive Value (PPV)**	**Negative Predictive Value (NPV)**
Radius	149.199	0.635	0.262	0.778	0.520	0.329	0.889	0.135
Radius UD	160.496	0.528	0.00	1.00	0.472	0.129	-	0.129
Radius 33%	10.942	0.716	0.459	0.667	0.563	0.486	0.903	0.154
Ulna	117.259	0.736	0.00	1.00	0.432	0.129	-	0.129
Ulna UD	162.088	0.643	0.705	0.556	0.630	0.686	0.915	0.217
Ulna 33%	73.365	0.708	0.00	1.00	0.454	0.129	-	0.129
Scaphoid	250.749	0.773	0.525	0.778	0.651	0.557	0.941	0.194
Lunate	258.091	0.768	0.00	1.00	0.433	0.129	-	0.129
Triquetrum	213.998	0.610	0.00	1.00	0.392	0.129	-	0.129
Pisiform	220.041	0.754	0.00	1.00	0.423	0.129	-	0.129
Trapezium	183.738	0.717	0.00	1.00	0.310	0.129	-	0.129
Trapezoid	269.594	0.726	0.656	0.778	0.717	0.671	0.952	0.250
Capitate	294.058	0.755	0.623	0.889	0.756	0.657	0.974	0.258
Hamate	171.503	0.673	0.00	1.00	0.423	0.129	-	0.129
1 MC	27.779	0.823	0.00	1.00	0.445	0.129	-	0.129
2 MC	30.584	0.752	0.721	0.889	0.805	0.743	0.978	0.320
3 MC	31.197	0.529	0.00	1.00	0.409	0.129	-	0.129
4 MC	55.376	0.579	0.770	0.556	0.663	0.743	0.922	0.263
5 MC	52.112	0.615	0.00	1.00	0.407	0.390	-	0.390
Linear kernel SVM		0.856	0.443	0.889	0.674	0.620	0.871	0.507
Radial basis function kernel SVM		0.969	0.885	0.667	0.805	0.800	0.806	0.788
Sigmoid kernel SVM		0.542	0.607	0.778	0.716	0.670	0.804	0.556
Random Forest classifier		0.511	0.967	0.222	0.595	0.680	0.663	0.818
**Femoral Neck BMD ≤ −2.5**	**Training/** **Validation** **Dataset CT Attenuation Threshold**	**AUC**	**Sensitivity**	**Specificity**	**AUC**	**Accuracy**	**Positive Predictive Value (PPV)**	**Negative Predictive Value (NPV)**
Radius	132.495	0.569	0.00	1.00	0.394	0.810	-	0.810
Radius UD	184.154	0.618	0.789	0.531	0.660	0.580	0.283	0.915
Radius 33%	20.908	0.625	0.00	1.00	0.426	0.810	-	0.810
Ulna	67.121	0.603	0.789	0.556	0.673	0.600	0.294	0.918
Ulna UD	82.730	0.581	0.526	0.790	0.658	0.740	0.370	0.877
Ulna 33%	35.520	0.621	0.00	1.00	0.375	0.810	-	0.810
Scaphoid	202.916	0.657	0.632	0.679	0.655	0.670	0.316	0.887
Lunate	224.838	0.684	0.526	0.864	0.695	0.800	0.476	0.886
Triquetrum	208.334	0.667	0.632	0.728	0.680	0.710	0.353	0.894
Pisiform	121.626	0.736	0.00	1.00	0.415	0.810	-	0.810
Trapezium	149.597	0.627	0.632	0.691	0.661	0.680	0.324	0.889
Trapezoid	207.953	0.663	0.632	0.679	0.655	0.670	0.316	0.887
Capitate	248.039	0.647	0.737	0.667	0.702	0.680	0.341	0.915
Hamate	185.743	0.600	0.842	0.568	0.705	0.620	0.314	0.939
1 MC	0.530	0.710	0.579	0.642	0.610	0.630	0.275	0.867
2 MC	−7.273	0.681	0.526	0.630	0.578	0.610	0.250	0.850
3 MC	−47.251	0.609	0.895	0.136	0.515	0.280	0.195	0.846
4 MC	−13.146	0.672	0.00	1.00	0.458	0.810	-	0.810
5 MC	24.690	0.737	0.00	1.00	0.398	0.810	-	0.810
Linear kernel SVM		0.915	0.947	0.593	0.795	0.660	0.535	0.980
Radial basis function kernel SVM		0.997	0.579	0.864	0.770	0.810	0.500	0.897
Sigmoid kernel SVM		0.736	0.947	0.531	0.749	0.610	0.321	0.977
Random Forest classifier		0.489	0.421	0.901	0.661	0.810	0.500	0.869
**Femoral Neck BMD < −1**	**Training/** **Validation** **Dataset CT Attenuation Threshold**	**AUC**	**Sensitivity**	**Specificity**	**AUC**	**Accuracy**	**Positive Predictive Value (PPV)**	**Negative Predictive Value (NPV)**
Radius	130.336	0.603	0.00	1.00	0.415	0.270	-	0.270
Radius UD	163.209	0.558	0.00	1.00	0.492	0.270	-	0.270
Radius 33%	10.942	0.605	0.00	1.00	0.423	0.270	-	0.270
Ulna	94.009	0.647	0.740	0.652	0.696	0.720	0.857	0.486
Ulna UD	185.544	0.684	0.00	1.00	0.363	0.270	-	0.270
Ulna 33%	27.406	0.618	0.727	0.739	0.733	0.730	0.883	0.500
Scaphoid	229.799	0.719	0.558	0.913	0.736	0.660	0.953	0.439
Lunate	268.193	0.707	0.00	1.00	0.331	0.270	-	0.270
Triquetrum	287.366	0.641	0.831	0.565	0.698	0.760	0.836	0.556
Pisiform	221.709	0.714	0.00	1.00	0.437	0.270	-	0.270
Trapezium	165.624	0.722	0.558	0.870	0.714	0.640	0.911	0.418
Trapezoid	236.041	0.693	0.610	0.696	0.653	0.640	0.849	0.404
Capitate	257.499	0.693	0.545	0.870	0.708	0.790	0.842	0.625
Hamate	160.072	0.584	0.00	1.00	0.299	0.270	-	0.270
1 MC	26.390	0.710	0.714	0.609	0.661	0.680	0.825	0.432
2 MC	9.576	0.700	0.623	0.870	0.746	0.680	0.918	0.451
3 MC	54.574	0.491	0.00	1.00	0.424	0.270	-	0.270
4 MC	5.199	0.616	0.00	1.00	0.427	0.270	-	0.270
5 MC	1.294	0.674	0.597	0.696	0.647	0.630	0.846	0.396
Linear kernel SVM		0.895	0.468	0.826	0.678	0.550	0.900	0.317
Radial basis function kernel SVM		0.987	0.584	0.957	0.818	0.670	0.978	0.407
Sigmoid kernel SVM		0.627	0.844	0.739	0.818	0.820	0.915	0.586
Random Forest classifier		0502	0.987	0.043	0.515	0.770	0.776	0.500

Radius—distal third of the radius; Radius UD—ultradistal radius (radius epiphysis/metaphysis); Radius 33%—distal third of the radial shaft; Ulna—distal third of the ulna; Ulna UD—distal ulna (ulnar epiphysis/metaphysis); Ulna 33%—distal third of the ulnar shaft; 1 MC—proximal third of the first metacarpal; 2 MC—proximal third of the second metacarpal; 3 MC—proximal third of the third metacarpal; 4 MC—proximal third of the fourth metacarpal; 5 MC—proximal third of the fifth metacarpal; -—Undefined.
