# Supplementary material for: Machine Learning for Opportunistic Screening for Osteoporosis from CT Scans of the Wrist and Forearm
Source: Diagnostics (Basel). 2022 Mar 11;12(3):691. doi: 10.3390/diagnostics12030691 (PMC8947723; doi:10.3390/diagnostics12030691)
Supplement: Supplementary file 1 [file diagnostics-12-00691-s001.zip › diagnostics-1626020-supplementary.pdf]

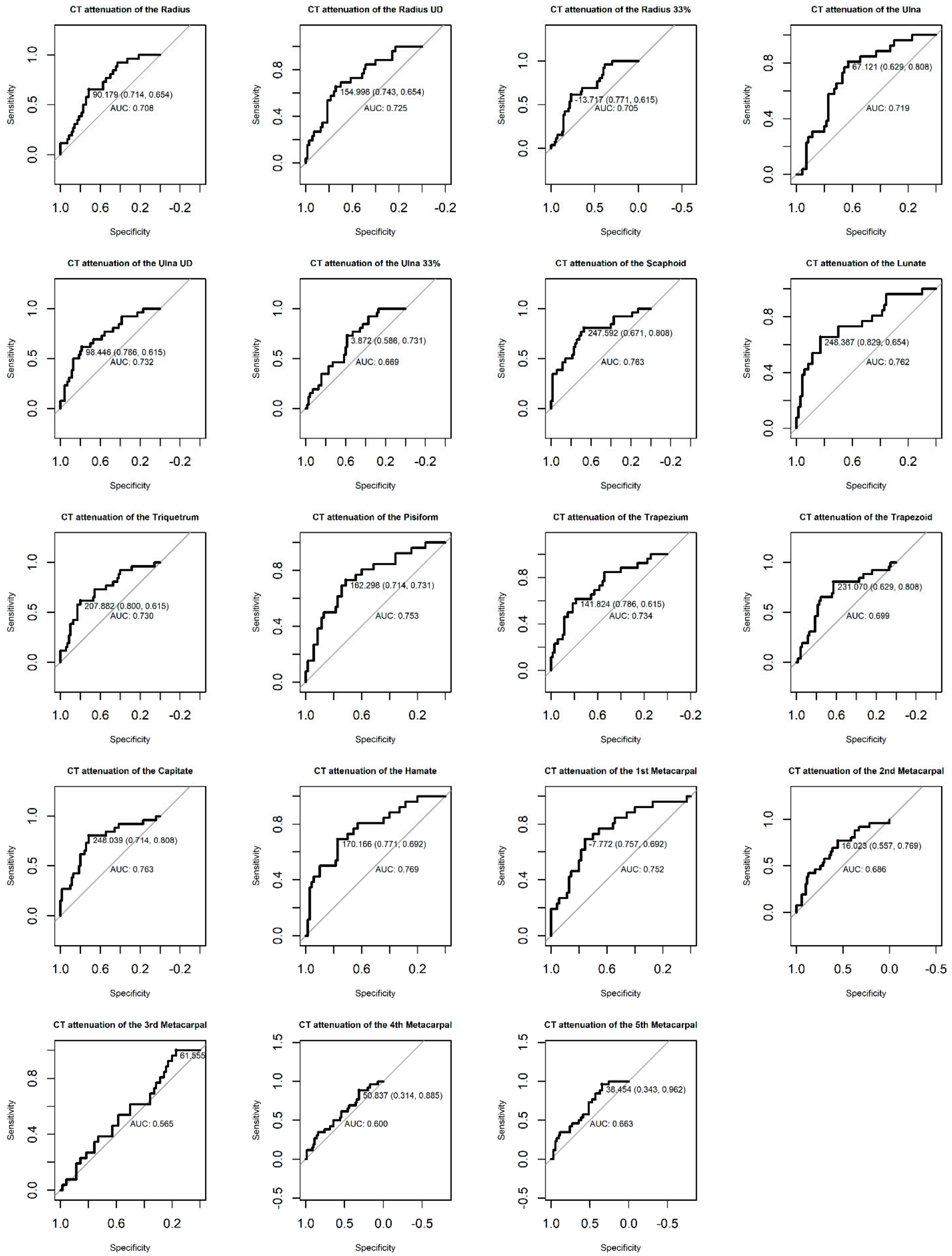

Figure S1. Performance of the CT attenuation of each bone and multivariable machine learning models to predict osteoporosis.

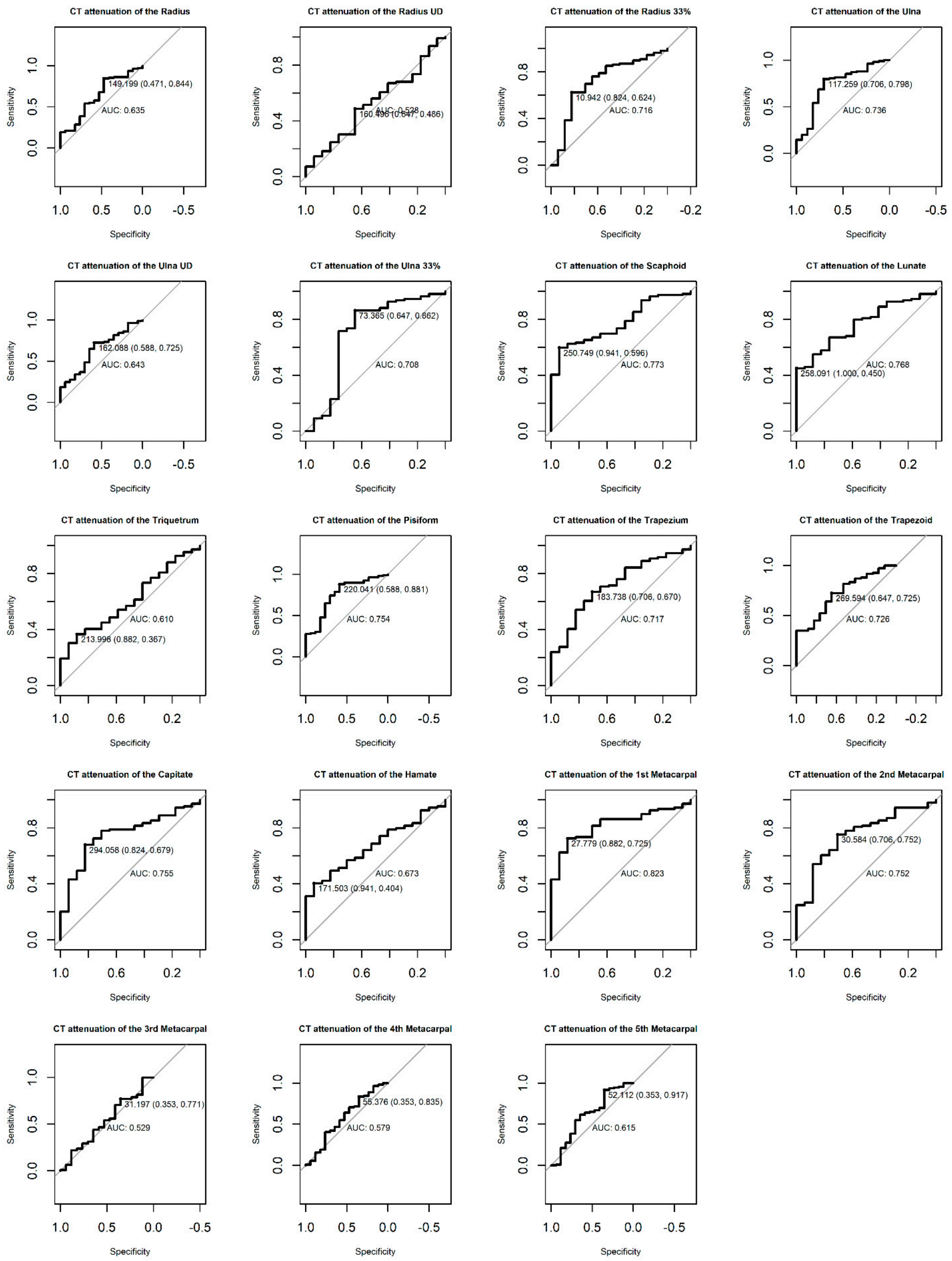

Figure S2. Performance of the CT attenuation of each bone and multivariable machine learning models to predict osteopenia/osteoporosis.

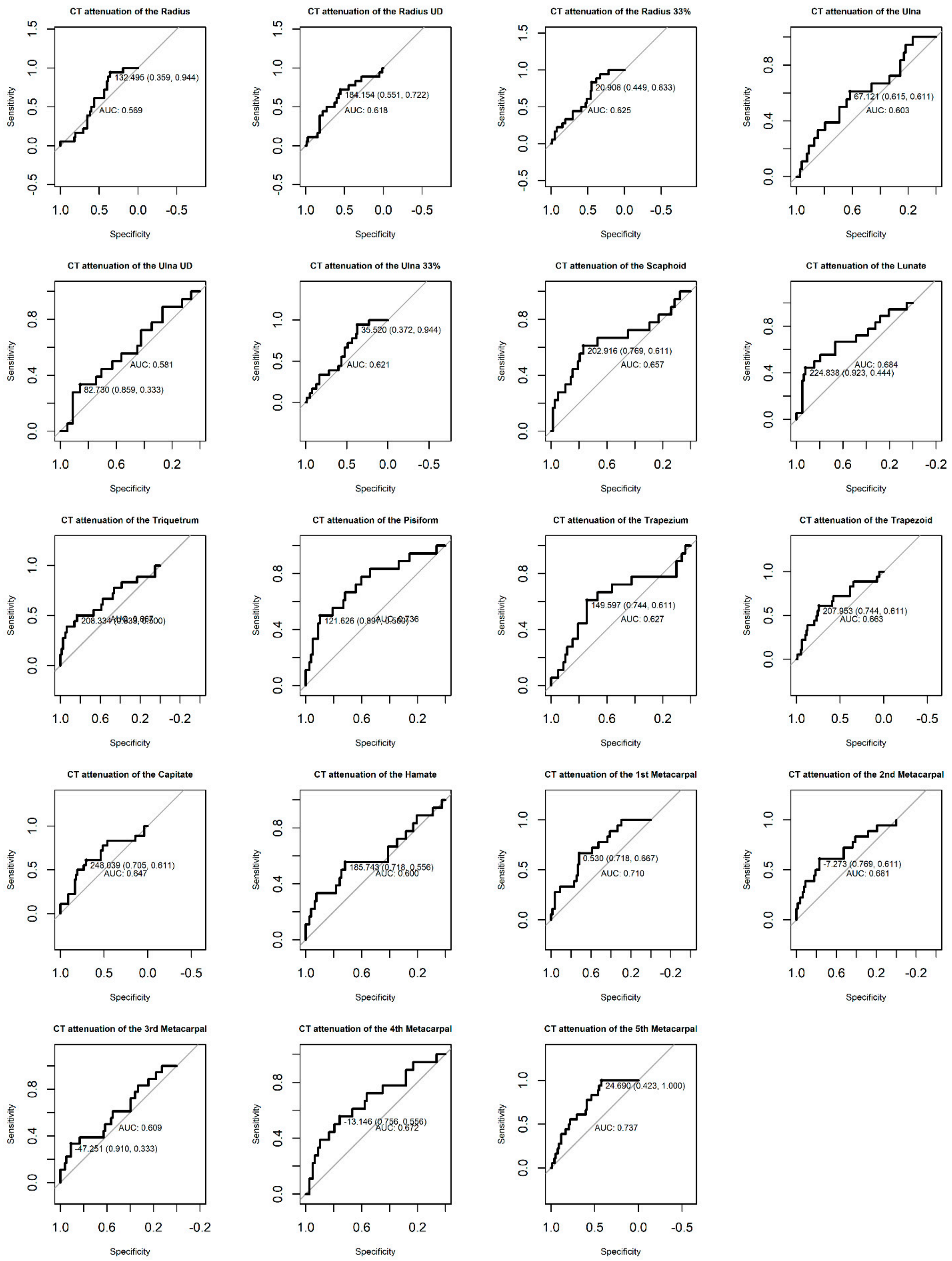

Figure S3. Performance of the CT attenuation of each bone and multivariable machine learning models to predict femoral neck BMD T-score ≤ −2.5.

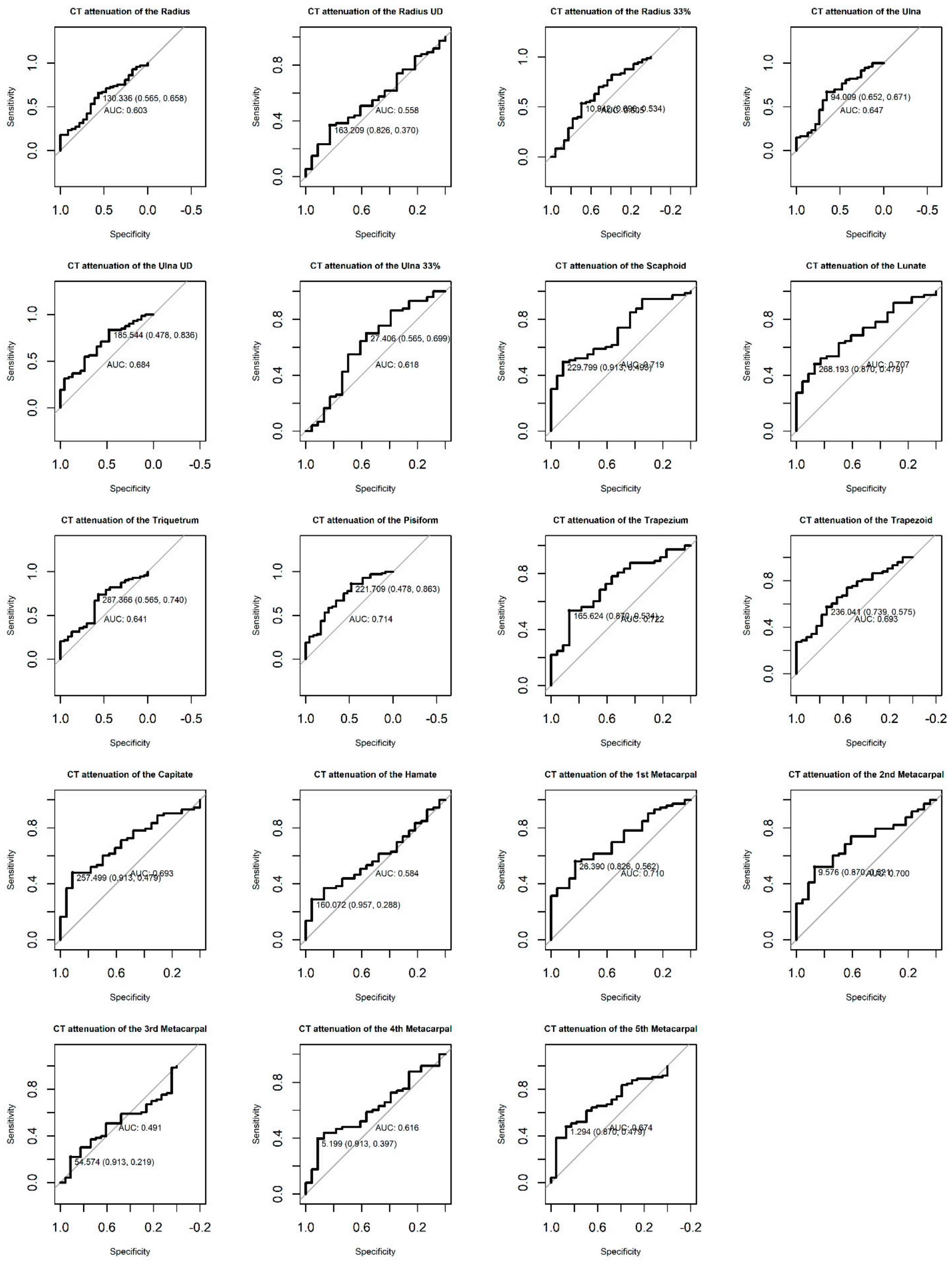

Figure S4. Performance of the CT attenuation of each bone and multivariable machine learning models to predict femoral neck BMD T-score < −1.
